# Association Between Acid-Sensing Ion Channel 3 Gene Variants and Balance Impairment in People With Mild Traumatic Brain Injury

**DOI:** 10.3389/fneur.2019.00088

**Published:** 2019-02-11

**Authors:** Hsun-Hua Lee, Hon-Ping Ma, Ju-Chi Ou, Jiann Ruey Ong, Kai-Yun Chen, Chung-Che Wu, Wen-Ta Chiu, Kuo-Hsing Liao, Chien-Min Lin, Shu-Yu Lin, Dean Wu, Yao-Hsien Huang, Yuan-Hung Wang, Chaur-Jong Hu, Chien-Tai Hong

**Affiliations:** ^1^College of Medicine, Graduate Institute of Clinical Medicine, Taipei Medical University, Taipei, Taiwan; ^2^Department of Neurology, Shuang Ho Hospital, Taipei Medical University, New Taipei City, Taiwan; ^3^Department of Neurology, School of Medicine, College of Medicine, Taipei Medical University, Taipei, Taiwan; ^4^Dizziness and Balance Disorder Center, Taipei Medical University–Shuang Ho Hospital, New Taipei City, Taiwan; ^5^Department of Emergency Medicine, Shuang Ho Hospital, Taipei Medical University, New Taipei City, Taiwan; ^6^Department of Emergency Medicine, School of Medicine, College of Medicine, Taipei Medical University, Taipei, Taiwan; ^7^College of Public Health and Nutrition, Graduate Institute of Injury Prevention and Control, Taipei Medical University, Taipei, Taiwan; ^8^College of Medical Science and Technology, Graduate Institute of Neural Regenerative Medicine, Taipei Medical University, Taipei, Taiwan; ^9^Department of Neurosurgery, Taipei Medical University, Taipei, Taiwan; ^10^Department of Neurosurgery, Wan Fang Hospital, Taipei Medical University, Taipei, Taiwan; ^11^Department of Neurosurgery, Shuang Ho Hospital, Taipei Medical University, New Taipei City, Taiwan; ^12^Graduate Institute of Biomedical Informatics, Taipei Medical University, Taipei, Taiwan; ^13^Sleep Center, Shuang Ho Hospital, Taipei Medical University, New Taipei City, Taiwan

**Keywords:** mTBI (mild traumatic brain injury), dizziness, balance function, ASIC3, stability, sensory integration

## Abstract

**Introduction:** Dizziness and balance impairment are common symptoms of mild traumatic brain injury (mTBI). Acid-sensing ion channel 3 (ASIC3) is expressed in the vestibular and proprioceptive systems and associated with balance functions. However, whether the genetic variants of *ASIC3* are associated with people who suffer dizziness and balance impairment after mTBI remained unknown.

**Materials and methods:** A total of 200 people with mTBI and 109 non-mTBI controls were recruited. Dizziness, balance functions, and the ability to perform daily activities were assessed by Dizziness Handicap Inventory (DHI), and objective balance functions were investigated by the postural stability test. Three diseases-related genetic variants of *ASIC3* were determined through polymerase chain reaction and followed by restriction fragment length polymorphism. The Student's *t*-test and Mann-Whitney *U*-test were used for normal and abnormal distributed data, respectively. The regression was applied to adjust gender and age. The normality of continuous data was evaluated by Shapiro-Wilk test.

**Results:** In the mTBI people, the rs2288645-A allele carriers exhibited a significantly worse physical domain DHI score (A-allele carriers: 11.39 ± 8.42, non-A carriers: 8.76 ± 7.87, *p* = 0.03). The rs4148855-GTC deletion carriers an exhibited significantly worse overall postural stability (GTC deletion carriers: 0.53 ± 0.33, non-carriers: 0.46 ± 0.20, *p* = 0.03). In the controls, rs2288646-A allele carriers were significant worse in the medial-to-lateral postural stability (A-allele carriers: 0.31 ± 0.17, non-A carriers: 0.21 ± 0.10, *p* = 0.01).

**Conclusion:** The present study demonstrated that *ASIC3* genetic variants were associated with certain aspects of balance functions and dizziness questionnaires in people of mTBI and non-mTBI. It provides a possible evidence that *ASIC3* could be a new target for the management of the balancing disorders. However, further investigations are warranted to elucidate the underlying mechanisms and clinical significance.

## Introduction

The incidence of mild traumatic brain injury (mTBI) is 100–300 per 100,000 people; however, if individuals who do not receive hospital treatment are considered, the actual incidence is likely doubled (1). Classical symptoms of mTBI include headache, sleep disturbance, and loss of concentration. Dizziness and balance impairment are also major symptoms of mTBI (2). Although most of these symptoms tend to partially resolve within 3 weeks (3), some chronic balance impairment after mTBI is detriment to the quality of life even years after the injury (4). Therefore, mTBI-related acute imbalance is a considerable and underacknowledged public health issue.

The maintenance of static balance necessitates coordination between the visual, vestibular, cerebellar, and proprioceptive nervous systems. Defects in any of them result in disequilibrium with corresponding presentations. For instance, vestibular dysfunction leads to spinning sensations, whereas the impairment of proprioceptive system causes imbalance exclusively in the dark without visual compensation (5). mTBI is known to cause traumatic injury-related vestibular diseases, including benign paroxysmal positional vertigo, labyrinthine concussion, and post-traumatic Ménière disease (6). In addition, mTBI damages the proprioceptive system (7) and the integrity of cerebellar white matter as well (8). The psychiatric symptoms of mTBI, such as anxiety and post-traumatic stress disorder, would further aggravate imbalance (9). People with mTBI were reported poor scores for the functional, emotional, and physical domains of the DHI and the stability and sway indices in postural stability test (10, 11).

The expression of acid-sensing ion channel 3 (ASIC3) is widely observed in both central and peripheral nervous systems, including the vestibular endorgans (12, 13), the dorsal root ganglion (14) and cerebrospinal fluid-contacting cells (CSF-c) as a motion and proprioceptive sensor (15). *ASIC3*-knockout mice were found to be defective in balance, including grid and beam walking (14). Genetic variants of *ASIC3*, in terms of rs2288645, rs2288646, and rs4148855, had been reported to be associated with human diseases, such as hypertension, pain, anxiety, and insulin resistance (16–18). Considering the prior researches had shown the genetic background could play important role in TBI outcome (19), the present study hypothesized that mTBI-related imbalance may be associated with genetic variants of *ASIC3*. The association between 3 possible pathological *ASIC3* genetic variants, rs2288645, rs2288646, and rs4148855 and the severity of balance impairment in people of mTBI were investigated by subjective questionnaires and an objective postural stability/sensory integration test. This study may help to understand the role of ASIC3 in dizziness/balance and individual differences in mTBI outcome and has the potential to lead to earlier and personalized interventions and rehabilitation.

## Materials and Methods

### Study Population

The present study was conducted in Shuang Ho Hospital, Taipei Medical University in New Taipei City, Taiwan during the period of time between June 2013 and December 2016. The inclusion criteria were modified from the definition of mTBI (20) as follows: (1) loss of consciousness for <30 min, (2) normal findings on computed tomography scans, and (3) a Glasgow coma scale score of 13–15. They were all at their 20–70 years old and got mTBI within 1 week. All the participants were comprised of healthy individuals with no known major diseases and had no any other type of head injury before. All the examinations were completed within 2 weeks after the onset of mTBI. All of the participants received history taking, detailed neurological examinations (including deep tendon reflex, cerebellar and proprioceptive assessments), and physical examinations by emergency physicians, neurosurgeons, and neurologists in emergency departments, inpatient and outpatient clinics. In addition to brain computed tomography, all the participants underwent brain magnetic resonance imaging to exclude brain, cerebellar, brainstem and vestibular structural abnormality or space occupied lesion. People were excluded if they had a history of epilepsy, cerebrovascular disease, unstable vital signs, pregnancy, uremia, heart disease, liver disease, alcoholism and illicit drug abuse, or any implant in body, including cochlear implant. This study was approved by the joint institutional review board of Taipei Medical University, and all the participants provided the written informed consent. In the control group, people were free from mTBI and selected by identical exclusion criteria as mTBI group. The recruitment was through in-house advertisements and most of them were employee or volunteer workers of the hospital.

### Dizziness Measurements

The severity of dizziness was assessed by the Dizziness Handicap Inventory (DHI). The DHI is a 25-item questionnaire that describes the physical, functional, and emotional aspects of daily living (21). Among the overall 25 set of questions, 9 sets are emotional related, 9 sets are functional related, and 7 sets are physical related questions. The DHI was a self-rating scale and recorded within 2 weeks post-mTBI.

### Postural Stability and Sensory Integration Tests

The postural stability test and the modified Clinical Test of Sensory Integration and Balance were performed using the Biodex Stability System (BSS; Biodex Medical Systems, Shirley, NY, USA). The BSS enables the objective assessment of neuromuscular control and somatosensory input (22) and consists of a moveable balance platform that provides up to 20° of surface tilt. Each participant was requested to achieve a comfortable foot position on the BSS platform while wearing shoes and maintain the same position during each testing session. The participants were instructed to maintain a forward-facing posture and hold their arms close to their torso during the measurements.

### Postural Stability Test

The postural stability test was used to assess balance functions according to three stability indices, including overall (OA), anterior-to-posterior, and medial-to-lateral stability. The stability indices were defined as the participant's average position relative to the center of the BSS platform, and therefore did not indicate his or her extent of swaying. When a participant was positioned on the BSS, such that his or her average position relative to the center was biased, the value of the stability index was high. This result for postural stability was calculated using a standard software configuration: a static platform in the anterior–posterior and medial–lateral axes and three 20-s trials along with a 10-s resting interval. Eight springs located under the outer edge of the platform provided resistance to the movement (i.e., the stability level of the platform). The resistance level of the platform was set to 8 (1, least stable; 8, most stable). These indices represented the fluctuations around a zero point, which was established on a stable platform surface before testing. In the postural stability test, a high OA stability index score indicated poor balance and stability.

### Modified Clinical Test of Sensory Integration and Balance

The modified Clinical Test of Sensory Integration and Balance provides a detailed and variable assessment on a static surface and consists of four sway indices for when participants stand on either a firm or foam surface with open or closed eyes. The average score was calculated for three tests among four sensory conditions: (1) eyes open while standing on a firm surface; (2) eyes closed while standing on a firm surface; (3) eyes open while standing on an unstable (foam) surface; and (4) eyes closed while standing on an unstable (foam) surface. A high sway index value indicates poor balance.

### Genomic DNA Extraction and Genotyping

Genomic DNA (gDNA) was extracted using the Blood Genomic DNA Extraction Midiprep System (Viogene, Sunnyvale, CA, USA). The *ASIC3* gene fragments were amplified through polymerase chain reaction (PCR) in a DNA thermal cycler (Perkin Elmer Cetus, Foster City, CA, USA) using the following forward (F) and reverse (R) primers for the alleles: for **rs2288645**, F: GAGGAGACCCCGTTTGAGGT and R: AGGCCCCTAGACAGGTCTGA; **rs2288646**, F: GCACCTGCTACCTTGTCACACAGCTCTAGA and R: GATGCATTAGGACTTTATTTGGGGTGGAGA; and **rs4148855**, F: CAGCCTCAGGCTGCCCAAAATGCCTCTGACACCAGATTT, and R: ACTCTCCACTGTGGAGCGAAGGCCGAGGCGAGGTTAGGGA. The PCR mixture (25 μL) contained 1 μg of gDNA, 100 ng of primers, 0.2 mM of each dNTP, 1 × PCR buffer, and 1 unit of Taq polymerase (Toyobo, Osaka, Japan). The reactions were performed for 35 cycles of 0.5 min at 94°C (denaturation), followed by 0.5 min at 59–62°C (annealing), and 30 min at 72°C (extension). The annealing temperatures varied with candidate fragments. After PCR amplification, 5 units of *AVAII* (for rs2288645) and *BsmBI* (for rs2288646 and rs4148855; New England Biolabs, Ipswich, MA, USA) were added to each reaction mixture for the digestion of these alleles at 37°C in a water bath for 2–3 h. The final products were loaded onto 2–3% agarose gels and stained with ethidium bromide for electrophoresis. The gel images were captured using the Image System (Fotodyne, Hartland, WI, USA), and the sizes of the digested fragments were determined on the basis of a comparison with a size marker (GeneDireX, Las Vegas, NV, USA). The allele frequencies and genotype distribution passed the Hardy–Weinberg equilibrium test.

### Statistical Analyses

The data were analyzed using the Student's *t*-test for normal distributed data and the Mann–Whitney *U*-test for abnormal data. The normality of continuous data was evaluated by Shapiro-Wilk test. The difference in DHI scores between two subgroups was evaluated via multivariate regression models along with adjusting age and gender. A *p*-value of < 0.05 was considered statistically significant. The R statistical software package (The R Foundation) was used for all statistical analyses.

## Results

Overall, 1,439 people met the inclusion criteria during the study period of time, and 243 participates agreed to join and signed the inform consent forms. Forty-three participates dropped out of this study and finally 200 patients completed the study. The baseline characteristics were not different between the participants who completed or dropped out of the study (data not shown). On the other hand, there were 109 controls enrolled. Table 1 demonstrated the demographic data of all study participants. There was no significant difference in either age and sex. Compared with controls, people with mTBI were significantly worse in dizziness and the balance function, assessed by either subjective DHI (total and three individual domains) or objective sensory integration on foam surface.

**Table 1:** Demographic data for mild traumatic brain injury (mTBI) and non-mTBI group.

	**mTBI, *n* = 200**	**Control, *n* = 109**	***P*-value**
Female	118	73	0.11
Age (year-old)	44.85 ± 13.80	46.34 ± 16.32	0.35
**INJURY MECHANISM(*****n*****)**			
Falls	56		
Traffic accident	84		
Others	60		
**DHI**			
Total	30.63 ± 25.49	10.49 ± 16.67	< 0.01^*^
Physical	9.76 ± 8.16	3.58 ± 4.43	< 0.01^*^
Emotional	8.53 ± 8.37	2.81 ± 5.97	< 0.01^*^
Functional	12.34 ± 11.00	4.11 ± 7.63	< 0.01^*^
**BALANCE—STABILITY**			
Overall	0.50 ± 0.28	0.47 ± 0.26	0.54
Anterior to posterior	0.38 ± 0.23	0.35 ± 0.24	0.13
Medial-to-lateral	0.21 ± 0.14	0.22 ± 0.11	0.06
**SENSORY INTEGRATION**			
EOFI	0.26 ± 0.26	0.52 ± 0.31	0.66
ECFI	1.13 ± 0.56	1.28 ± 0.80	0.31
EOFO	0.95 ± 0.50	0.78 ± 0.22	< 0.01^*^
ECFO	3.18 ± 1.28	2.56 ± 0.98	< 0.01^*^

*n: count, DHI, Dizziness Handicap Inventory; EOFI, eyes open while standing on a firm surface; ECFI, eyes closed while standing on a firm surface; EOFO, eyes open while standing on an unstable (foam) surface; ECFO, eyes closed while standing on an unstable (foam) surface*,

* *indicates p < 0.05; The data were analyzed using the Chi-squared test for injury mechanism and the Mann–Whitney U test for the others data, and presented by (mean ± standard deviation)*.

Table 2 showed the proportion of the *ASIC3* variants. Three genetic variants of *ASIC3*, namely rs2288645, rs2288646, and rs4148855, were analyzed. The alleles, rs2288645, rs2288646, and rs4148855, had Hardy–Weinberg Equilibrium *p*-values of 0.16, 0.69, and 0.35, respectively.

**Table 2:** The proportion of three *ASIC3* genetic variants among mild traumatic brain injury (mTBI) and non-mTBI group.

	**mTBI, *n* = 200**	**Control, *n* = 109**	***P*-value**
rs2288645			0.84
AA	5	4	
AG	71	38	
GG	124	67	
rs2288646			0.12
GG	189	97	
AG	11	12	
AA	–	–	
rs4148855			0.08
–/–	17	7	
–/GTC	92	38	
GTC/GTC	91	64	

*The count data were analyzed by using the Chi-squared test*.

The effect of *ACIS3* genetic variants on subjective post-mTBI dizziness was assessed by DHI. The three *ASIC3* genetic variants did not have significant impact on total and individual domain of DHI scores in control group. In contrast, among mTBI group, rs2288645-A allele carriers conferred higher score in the physical domain of DHI (A-allele carriers: 11.39 ± 8.42, non-A carriers: 8.76 ± 7.87, *p*-value = 0.03 after adjusting age and gender). The rest of two genetic variants, rs2288646 and rs4148855, had no significant influence on the overall and individual domain scores of the DHI compared between minor with the major allele carriers (Table 3).

**Table 3:** Comparison of DHI, postural stability and sensory integration between the ASIC3 genetic variants.

		**DHI-physical**		**DHI-emotion**		**DHI-function**		**Balance—stability -Overall**		**Balance—stability anterior-to-posterior**	
		**mTBI**	**Control**	**mTBI**	**Control**	**mTBI**	**Control**	**mTBI**	**Control**	**mTBI**	**Control**
rs2288645	AA&AG	11.39 ± 8.42^*^	2.76 ± 3.87	8.74 ± 9.25	2.24 ± 5.41	13.47 ± 11.52	2.81 ± 5.25	0.49 ± 0.26	0.50 ± 0.22	0.38 ± 0.20	0.37 ± 0.20^*^
	GG	8.76 ± 7.87	4.09 ± 0.71	8.40 ± 7.81	3.16 ± 6.31	11.65 ± 10.66	4.93 ± 8.73	0.50 ± 0.29	0.45 ± 0.28	0.39 ± 0.25	0.33 ± 0.26
rs2288646	AG	13.09 ± 9.97	1.33 ± 3.23	9.64 ± 9.79	0.5 ± 1.73	15.45 ± 13.92	1.33 ± 3.23	0.48 ± 0.29	0.57 ± 0.31	0.35 ± 0.20	0.39 ± 0.27
	GG	9.57 ± 8.03	3.86 ± 4.49	8.47 ± 8.30	3.09 ± 6.25	12.16 ± 10.83	4.45 ± 7.95	0.50 ± 0.28	0.45 ± 0.25	0.38 ± 0.24	0.34 ± 0.23
rs4148855	(–/–)&(–/GTC)	9.47 ± 8.12	3.6 ± 4.56	8.26 ± 8.13	3.42 ± 8.01	11.65 ± 10.56	3.91 ± 8.23	0.53 ± 0.33	0.49 ± 0.35	0.41 ± 0.28	0.36 ± 0.33
	(GTC/GTC)	10.11 ± 8.24	3.56 ± 4.37	8.86 ± 8.67	2.38 ± 3.98	13.16 ± 11.51	4.25 ± 7.23	0.46 ± 0.20	0.45 ± 0.16	0.35 ± 0.16	0.34 ± 0.15
		**Balance—stability medial-to lateral**		**Sensory integration-EOFI**		**Sensory integration-ECFI**		**Sensory integration-EOFO**		**Sensory integration-ECFO**	
		**mTBI**	**Control**	**mTBI**	**Control**	**mTBI**	**Control**	**mTBI**	**Control**	**mTBI**	**Control**
rs2288645	AA&AG	0.21 ± 0.15	0.24 ± 0.13	0.52 ± 0.22	0.52 ± 0.22	1.07 ± 0.48	1.27 ± 0.76	0.92 ± 0.36	0.79 ± 0.21	3.06 ± 1.12	2.71 ± 0.98
	GG	0.21 ± 0.14	0.21 ± 0.10	0.53 ± 0.28	0.52 ± 0.36	1.16 ± 0.60	1.29 ± 0.83	0.97 ± 0.56	0.77 ± 0.23	3.25 ± 1.37	2.46 ± 0.97
rs2288646	AG	0.23 ± 0.17	0.31 ± 0.17^*^	0.50 ± 0.30	0.58 ± 0.17	0.94 ± 0.34	1.33 ± 0.50	0.89 ± 0.18	0.84 ± 0.20	3.02 ± 1.07	2.72 ± 0.74
	GG	0.21 ± 0.14	0.21 ± 0.10	0.53 ± 0.26	0.51 ± 0.33	1.14 ± 0.56	1.28 ± 0.83	0.95 ± 0.51	0.77 ± 0.22	3.19 ± 1.30	2.54 ± 1.00
rs4148855	(–/–)&(–/GTC)	0.22 ± 0.16	0.24 ± 0.13	0.55 ± 0.30	0.56 ± 0.42	1.10 ± 0.56	1.42 ± 0.95	1.00 ± 0.58	0.77 ± 0.25	3.27 ± 1.38	2.56 ± 1.00
	(GTC/GTC)	0.20 ± 0.12	0.21 ± 0.10	0.50 ± 0.19	0.50 ± 0.20	1.16 ± 0.55	1.18 ± 0.67	0.89 ± 0.38	0.78 ± 0.20	3.07 ± 1.16	2.56 ± 0.96

*DHI, Dizziness Handicap Inventory; EOFI, eyes open while standing on a firm surface; ECFI, eyes closed while standing on a firm surface; EOFO, eyes open while standing on an unstable (foam) surface; ECFO, eyes closed while standing on an unstable (foam) surface*,

* *indicates p < 0.05; The data were presented by (mean ± standard deviation)*.

Regarding to the objective assessments of balance function, all study participants were undergoing postural stability and sensory integration examinations. Revealed by Table 3, genetic variants rs2288645 did not have influence on the overall and individual parameter of the postural stability assessment in either mTBI or control group. *ASIC3* rs4148855-GTC deletion was associated with significantly higher overall scores in the postural stability assessment (GTC deletion carriers: 0.53 ± 0.33, non-carriers: 0.46 ± 0.20, *p* = 0.03 after adjusting age and gender), which indicated worse objective balance function in the people with mTBI. The rs2288646 A-allele carrier had significant worse postural stability in the medial-to-lateral domain among control group (A-allele carriers: 0.31 ± 0.17, non-A carriers: 0.21 ± 0.10, *p* = 0.01 after adjusting age and sex) but not people with mTBI. Lastly, we assessed the sensory integration in both groups to evaluate. After adjusting age and gender, there was no significant difference in all sensory integration conditions between all three *ASIC3* genetic variants in either mTBI or control group.

## Discussion

Although genetic polymorphisms or variants had been reported to influence several outcomes of mTBI, such as catecholamine and neurotrophic genes (23), none of these documented variants were associated with balance deficit (24). The present study demonstrated that *ASIC3* genetic variants were associated with balance impairment and dizziness for people with mTBI. To our knowledge, the present study is the first to investigate the specific association between *ASIC3* genetic variants and balance functions in people with mTBI and normal population.

These genetic variants of *ASIC3*, rs2288645, rs2288646, and rs4148855 had been reported to be associated with human diseases, such as hypertension, pain, anxiety, and insulin resistance (16–18). The present study found that the rs2288645-A allele carriers had significantly poorer physical domain scores in the DHI, and the rs4148855-GTC deletion carriers exhibited poorer overall scores in the postural stability test after mTBI. In addition, rs2288646 A-allele carrier had significant worse postural stability in the medial-to-lateral domain at their baseline. The discrepancy may result from the different location of the variants, which affects the expression or function of ASIC3 in distinct manners. The rs2288645 allele was located in the intron region, and the rs2288646 and rs4148855 alleles were located in the 3′ untranslated regions. Although these variants in the present study did not directly alter the amino acid sequence of the ASIC3 protein, both rs2288646 and rs4148855 are located at the DNA binding sites for transcription factors and could influence transcriptional regulation (25). Therefore, the changes in alternative splicing or other molecular regulatory mechanisms due to these *ASIC3* genetic variants may result in functional differences. In addition, the discrepancy was also possibly because of the complexity of the mTBI-related disequilibrium. Unlike other pure vestibular or cerebellar disorders, balance impairment is one of the numerous symptoms of mTBI (26, 27). Dizziness and vertigo may directly result from trauma to the vestibular and central nervous systems or were secondary to psychiatric symptoms of mTBI, such as anxiety, depression, or post-traumatic stress disorder (6–9). The mixture of neurological and psychiatric symptoms may affect the sensitivity and specificity of the DHI and other balance test in different scales.

The outcomes of mTBI are associated with many factors, including genetic factor (28), the personal factors, types of injury and the acute consequences. Female and old age are the major personal risk factors for poor prognosis of mTBI. People aged below 50 years old are at a lower risk of post-mTBI imbalance (29). In animals with repetitive mTBI, the post-traumatic behavioral changes and neuroimages are different among males and females (23). The present study demonstrated that rs2288645 and rs4148855 were associated with subjective balance function questionnaires and objective postural stability tests after adjusting age and sex. These findings indicated that genetic variants of *ASIC*3 could be an independent factor and might play an important role in post-mTBI balance functions.

ASIC3 is an ion channel that was initially identified to respond to the changes in pH and mechanical stimulation (30–33). Acidification generates action potentials in the vestibular neurons, whereas the ASIC antagonists, amiloride and acetylsalicylic acid, significantly reduce the vestibular nerve discharge (12, 13). The molecular topology of ASICs is similar to the mechanosensory proteins expressed in nematodes touch receptor neurons and involved in neurosensory mechanotransduction (25). Recently, ASIC3 on the dorsal root ganglion was reported to be involved in the proprioceptive and balance functions in mouse models (14). The mice with floxed allele of *ASIC3* were defective in grid and balance beam walking tasks (14). Moreover, *ASIC3* expression was observed in the vestibular endorgans, which is responsible for balance functions. Another speculated linking between *ASIC3* and the recovery of balance impairment after mTBI was that pressure pulse activates ASIC3 of ciliated cerebrospinal CSF-c (15). Impact induced by mTBI may have effects on ASIC3 in inner ears is the possible mechanism, because endolymph-contacting ciliated hair cells were similar to CSF-c in morphology and functions (15, 34). Considering the possible role of ASIC3 in the balance function, the modulation of the ASIC3 may improve the balance function of people with mTBI. Recently, there was a new peptide, APETX2 can specifically inhibit ASIC3 (35). With the development of other novel agents to manipulate this channel, people are able to avoid the profound imbalance sequalae after mTBI in the future.

The strength of the present research is that the genetic variants of ASIC3 are associated with imbalance in people of mTBI. The results of the present study may contribute to the further understanding of disease mechanisms and potential treatment targets of post-concussion dizziness and balance impairment. Nevertheless, this study had some limitations. First, there was no direct comparison between before and after mTBI in the same individual. Since the mTBI is mainly unpredictable, it is not likely to obtain this pre-mTBI information before enrollment. The present study utilized healthy controls to severe as the baseline of the balance function. It would be even better to recruit people with general trauma to serve as a disease control for avoiding the bias from the trauma-related psychiatric problems. However, it would introduce other confounding factors, like the difficulty to perform the sensory integration assessment due to the physical discomforts after trauma. Second, the present study did not perform the electronystagmography, caloric testing, rotatory chair, and other vestibular tests. These assessments can be used to confirm the possible vestibular dysfunction but were time consuming. In order to avoid the high drop-out rate, therefore, we used DHI and postural stability test to evaluate the participants' vestibular function (36). These assessments evaluated the overall balance function, rather than purely cerebellar or vestibular function. Lastly, the present study did not evaluate the physiological and pathological roles of ASIC3 in balance functions, which were required more studies to investigate.

In conclusion, *ASIC3* genetic variants were associated with balance impairment and dizziness among normal population and people of mTBI. *ASIC3* genetic variants may affect the clinical manifestations of the mTBI and are likely to be a new target for management the imbalance after mTBI. Further studies on the pathophysiological effects of ASIC3 on mTBI-induced balance impairment and dizziness are warranted.

## Ethics Statement

This study was carried out in accordance with the recommendations of JIRB-TMU with written informed consent from all subjects. All subjects gave written informed consent in accordance with the Declaration of Helsinki. The protocol was approved by the JIRB-TMU.

## Author Contributions

H-HL, C-JH, and C-TH mainly wrote the article. K-YC, C-CW, W-TC, K-HL, C-ML, and S-YL designed the study. H-PM, JCO, and JO performed the analytic calculation. K-YC, C-CW, and Y-HW verified the analytic methods. All authors discussed the result and commented on the manuscript, and all authors contributed to the final version of the manuscript.

### Conflict of Interest Statement

The authors declare that the research was conducted in the absence of any commercial or financial relationships that could be construed as a potential conflict of interest.
